# Effect of prenatal methamphetamine administration during gestational days on mice

**Published:** 2015-01

**Authors:** Arezoo Khoradmehr, Amirhossein Danafar, Iman Halvaei, Jalal Golzadeh, Mahya Hosseini, Tahereh Mirjalili, Morteza Anvari

**Affiliations:** 1 *Research and Clinical Center for Infertility, Shahid Sadoughi University of Medical Sciences, Yazd, Iran.*; 2 *Young Researchers Club, Islamic Azad University, Yazd, Iran.*; 3 *Department of Biology, College of Sciences, Shiraz University, Shiraz, Iran.*; 4 *Department of Biology and Anatomy, Shahid Sadoughi University of Medical Sciences, Yazd, Iran.*

**Keywords:** *Pregnant mice*, *Methamphetamine*, *Embryo development*, *Apoptosis*

## Abstract

**Background:**

Methamphetamine (MA) is one of most common illicit drugs which were reported that nearly half of MA consumers are women. MA can cross through placenta and affects pregnancy and fetus development.

**Objective:**

Our aim was to evaluate effects of injected MA on crown-rump length, head and placental circumference, body weight, histological changes and apoptosis in fetus.

**Materials and Methods:**

Twenty-four NMRI pregnant mice were randomly divided into five groups. First, second and third groups were injected intraperitoneally 10 mg/kg/day MA during gestational days (GD): GD1-7, GD8-14, and GD1-14, respectively. Forth group, as sham, was injected saline from GD1-14, and finally control which was received neither MA nor saline. On GD15 cervical dislocated pregnant mice, fetus and placenta were weighed and fetus crown-rump length was measured. Hematoxylin and Eosin staining and TUNEL assay were applied to assess histological changes and apoptosis respectively.

**Results:**

Fetus body weight and crown-rump length showed significant decrease in third compared to first and second groups (p≤0.001). There were significant differences in head circumference in control and sham compared to third group (0.5 (0.5-0.6), 0.6 (0.5-0.8), 0.4 (0.4-0.5) cm respectively, p≤0.001). Also fetus that treated with MA showed lower placenta circumference compared to control and sham groups. Histological changes such as exencephaly, hemorrhage and immature fetus were observed in second and third groups. Apoptotic cells in second and third groups were higher than controls, but differences were not significant.

**Conclusion:**

It seems MA abuse during pregnancy can cause morphological and histological changes in mice fetus but the exact mechanism remains unclear.

## Introduction

Percentage of the women who are in reproductive age and use illicit drugs is growing and while of illicit drugs affects pregnant women and fetal development (1, 2). Meanwhile, methamphetamine (MA) is one of the most common illicit drugs, it was reported that nearly half of the MA consumers are women (3). Recreational drugs can have different side effects during different pregnancy trimesters (4). Animal studies suggest that MA abuse in first and third trimester can generate long-term effects in dopamine and serotonin systems and impact on learning and social development of human fetus (4). It was shown that, MA has neurotoxic effects on central nervous system (CNS) by increasing dopamine and norepinephrine that inhibiting monoamine oxidase may leading to enhance synaptic catecholamine levels (5). 

The metabolites of dopamine and norepinephrine induce oxidative stress which leads to DNA damages (6). MA use also may lead to hyperreflexia, irascibility, confusion, aggressiveness, and pursued by weariness and depression (7). MA cause maternal depression and anxiety before, during and after pregnancy that realize high influence on fetus development (4). Gestational days 8-15 of a 20-days pregnancy are a critical time in mice that organogenesis occurs (8). MA can cross the placenta and may have detrimental impacts on the fetus and related tissues (9). Teratogenic agent usage during pregnancy leads to fetal death and structural abnormalities such as cleft palates and exencephaly (8). In utero administration of MA induces calcification and morphological damage of the placenta as well as abortion and maternal death (10). 

Some effects of prenatal MA exposure on the fetus are reduction of birth weight and head circumference, cerebral hemorrhage, growth retardation, prematurity, cardiac anomalies, cleft palate and possibly death (11, 12). MA may affect liver, brain, and cardiovascular systems. Also it can cause hyperthermia and even sudden death in consumers. High body temperature following MA abuse can have negative results on pregnancy outcomes and fetus development (13). 

MA has direct cardiovascular effects, causes hypertension and vasoconstriction (14). Prematurity, placental hemorrhage and spontaneous abortion suggested that MA exposure perhaps can intensifies uterine and placenta vasoconstriction and on the other hand, reduces uterine blood flow which provokes fetal hypoxemia and hypertension (15). Also MA is considered as reactive oxygen species (ROS) initiating teratogenic agents. In spite of difficulties to explain the direct mechanism of teratogenesis during organogenesis period, it has been proved that the levels of most antioxidants are low in the embryos and proceed them to be more susceptible to ROS elevation. 

ROS can deregulate signal transduction and cause molecular oxidative damages such as lipids, proteins, DNA, and likewise triggers apoptotic or necrotic cell death (8). Some evidences support that MA enhances hydroxyl radical formation and oxidation of embryonic proteins. Hydroxyl radicals cause DNA damage and participate in formation of 8-hydroxyguanine and its keto-form, 7,8-dihydro-8-oxoguanine (8-oxoG)(16). 

Enhancement of 8-oxoG levels by MA in embryo which was illustrated by Jeng *et al* can affect the expression and activity of proteins that are required for normal development of fetus and its function (16). In contrast, animal studies have shown that MA can have detrimental effects on fetus developing and there is few data in utero impact on human fetus. There are few studies regarding the probable effects of MA on fetus and little is known about how MA disrupts the fetus. To the best of our knowledge, there is no published study focusing exactly on prenatal administration of MA in first and second weeks of pregnancy.

Here, our main goal was to evaluate the effect of prenatal exposure of 10 mg/kg/day of MA in pregnant mice on different days of pregnancy on crown-rump length, placental circumference, body and placental weight gain of fetus and histological changes as well as mother weight gain during MA administration.

## Materials and methods

**Methamphetamine and animals treatment**

In this experimental study, 8-12 weeks old NMRI female mice, one week before mating, were acclimatized to an almost constant temperature of 23±2^o^C with a relative humidity of 55±5%, light-dark cycle of 12:12 hr, food and water which were provided ad libitum, in plastic cages. The use of animals was according to guide for the care and use of laboratory animals and this study was approved at our institute ethics committee of Research & Clinical Center for Infertility, Shahid Sadoughi University of Medical Sciences, Yazd, Iran. 

Females were housed with a male for one night. The next morning vaginal plug observation was considered as GD1. Pregnant mice were divided into five groups. Three MA treated groups which were injected 10 mg/kg/day of MA respectively, from GD1 to GD7, GD8 to GD14 and GD1 to GD14. Sham group was injected saline and fifth group as control. Body weight of the pregnant mice was measured on GD1 and GD15 in all groups. On GD15, the pregnant females were killed by cervical dislocation, and then their fetuses were removed by cesarean section. Fetus and placenta were weighed and finally fixed in 7% formaldehyde at 4^o^C. Also abortion and birth rates were reported in each group. Crown-rump length, head and placenta circumference of fetus were measured with caliper. The head circumference was measured with C=π {3(A+B)-[(3A+B) (A+3B)] 1/2} formula.

**Histological evaluation**

Fixed fetus and placenta were dehydrated with ethanol series, embedded in paraffin and then sectioned at 5µm thickness. Sections were stained by Hematoxylin and Eosin and histological changes were evaluated using light microscope (17). TUNEL assay was used to detect cell apoptosis. To achieve this aim, fetus brain was dewaxed in xylene and rehydrated through a graded series of ethanol. Then, the samples were washed 30 min with PBS and incubated in 0.1% Triton X-100 for two min on ice (2-8^o^C). After that, the slides were incubated in 50 µl TUNEL (In Situ Death Detection Kit Fluorescein, Roche, Germany) reaction mixture for 60 min at 37^o^C in the dark room (according to manufacturer’s instructions). Just then, sample analysis was performed under a fluorescence microscope (Olympus BX51, Japan). Slides were incubated in 50µl label solution instead of TUNEL reaction mixture for negative control and DNase I grade I (3 U/ml in 50 mM Tris-HCl, pH 7.5, 1 mg/ml BSA) was used for 10 min in order to determine positive control.

**Statistical analysis**

The data were expressed as median (min-max). Kruskal-Wallis test was used to compare between different groups. The level of significance was determined to be at p≤0.05. 

## Results

**Maternal and fetus body and placenta weight gain**

**Figure 1:**
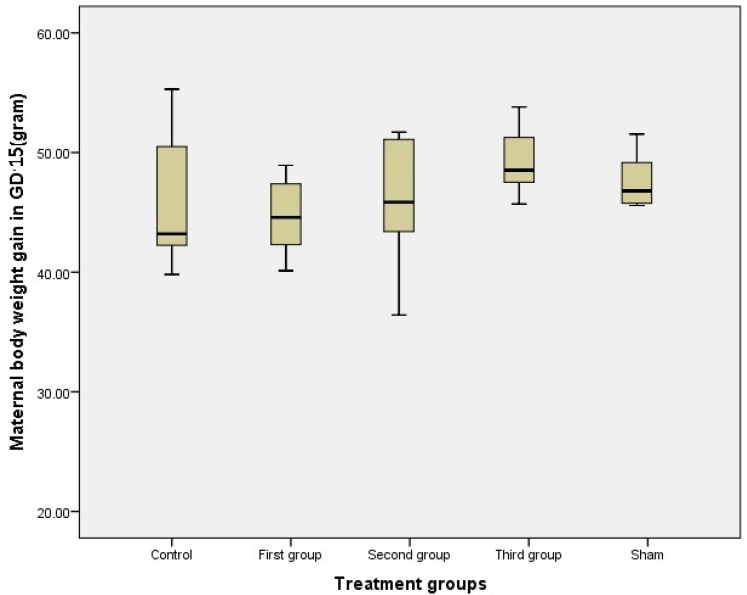
Maternal body weight gain between groups; data show administration of MA in different weeks of pregnancy has no effect on body weight gain in mothers in treated, sham and control groups (first, second and third groups were injected 10mg/kg/day of MA respectively, from GD1 to GD7, GD8 to GD14 and GD1 to GD14).

Administration of MA did not affect mother body weight gain (Figure 1). Lower body weight gain in fetus was observed in second and third groups compared to the controls and shams (p≤0.001) and also, there was a significant decrease in second and third groups but not in first group during MA administration (Table I). In addition the placenta weight gain was decreased in second and third groups compared to other groups (p≤0.001) (Table I).

**Head and placenta circumference and crown-rump length in fetus**

According to table I, all treated groups showed decreasing trend in head circumference in comparison with the controls and shams (p≤0.001). There was lower fetus placenta circumference among MA treated and controls (p≤0.001) (Table I). Although, significant reduction of crown-rump length was observed in treated groups compared to control and sham groups (p≤0.001) (Table I).

**Abortion and birth rate**

**Figure 2:**
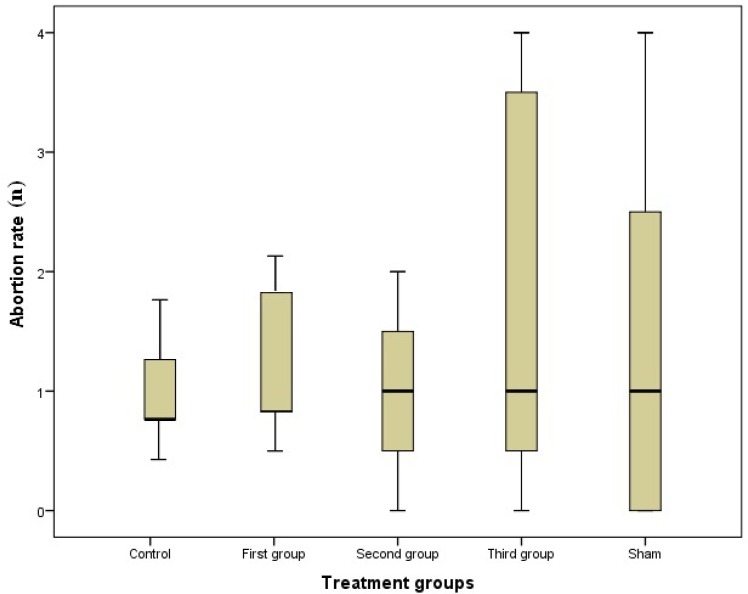
Abortion rate in groups; there is no significant different in treated groups compared to control and sham groups (first, second and third groups were injected 10mg/kg/day of MA respectively, from GD1 to GD7, GD8 to GD14 and GD1 to GD14).

**Figure 3:**
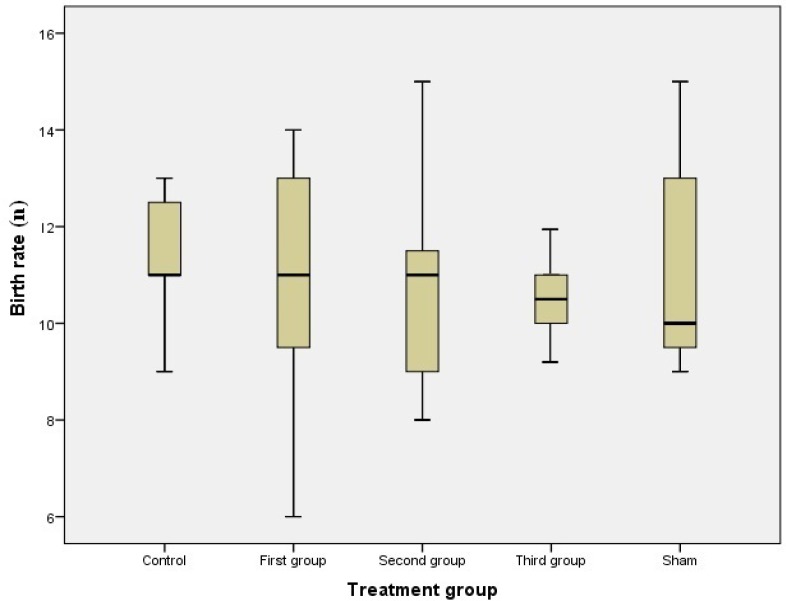
Birth rate in different groups; administration of MA determined that there is no difference on birth rate between treated groups compared to control and sham groups (first, second and third groups were injected 10mg/kg/day of MA, from GD1 to GD7, GD8 to GD14 and GD1 to GD14 respectively).

The data showed that different rates of abortion were gained in all groups, but the differences were insignificant (Figure 2). Birth was also observed in treated, control and sham groups, but there was pointless difference when it was compared between groups (Figure 3).

**Histological alterations**

**Figure 4:**
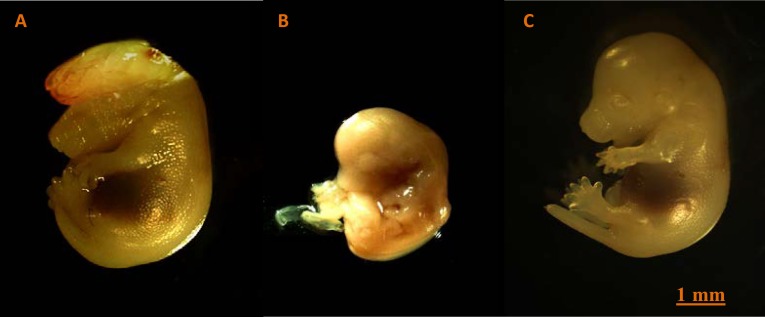
Mouse fetus after 15 days of MA exposure illustrates the A: Exencephaly in third group, B: Immature fetus in third group, C: Normal embryo (Magnification 10x)

**Figure 5:**
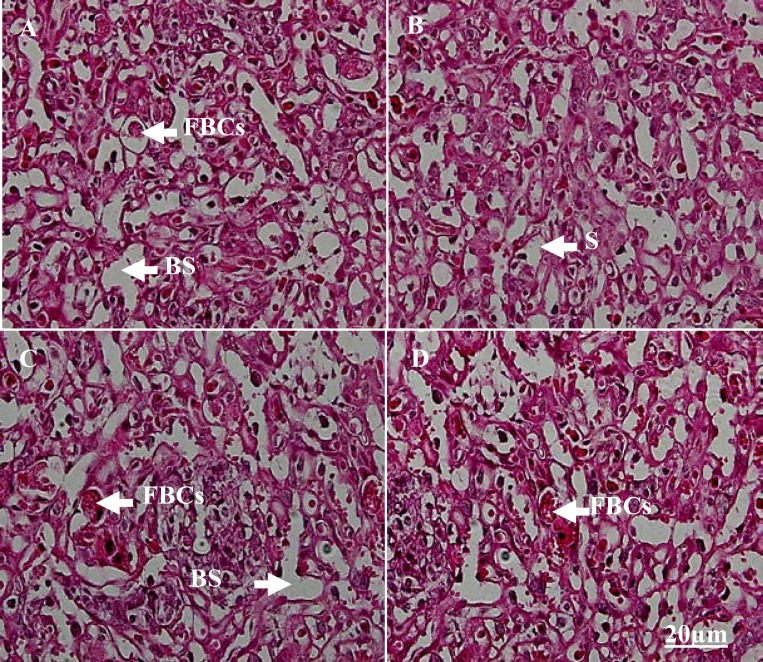
Placenta sections in control (A), first (B), second (C) and third groups (D).There were no calcification and morphological damages in placenta. FBCs: fetal blood cells, BS: blood space, S: syncytiotrophoblast (Magnification 400x)

**Figure 6:**
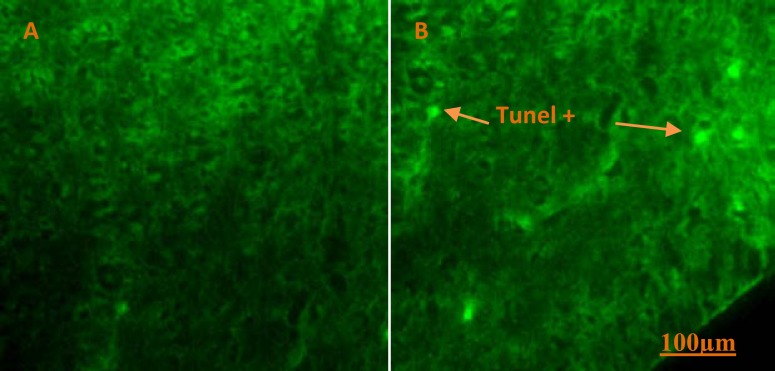
TUNEL assay in control (A) and third groups (B); positive apoptosis (Green point) marked by orange arrow, abundance of apoptotic cells in second and third groups (Magnification 200x).

Prenatal MA exposure was lead to produce fetus with exencephaly (Figure 4-A) and subarachnoid hemorrhage in third group. Also immature fetus in third group was observed (Figure 4-B) but no calcification and morphological damage in placenta was notified (Figure 5). There was increasing trend in apoptotic cells in second and third groups compared to the other groups (Figure 6).

**Table I:** Body weight gain, crown-rump length, head and placenta circumference, placenta weight in different groups

** Groups**	**Control**	**Sham**	**First group**	**Second group**	**Third group**
**Parameters**					
Body weight gain (gr)	0.02 (0.02-.03)	0.02 (0.02-0.03)	0.02 (0.01-0.04)	0.02 (0.01-0.04)*	0.02 (0.009-0.04)*
Placenta weight (gr)	0.01 (0.008-0.02)	0.01 (0.007-0.02)	0.01 (0.009-0.02)	0.01 (0.004-0.01)*	0.01 (0.004-0.02)*
Head circumference (cm)	0.5 (0.5-0.6)	0.6 (0.5-0.8)	0.5 (0.4-0.5)*	0.48 (0.4-0.5)*	0.4 (0.4-0.5)*
Placenta circumference (cm)	0.8 (0.7-0.9)	0.8 (0.6-0.9)	0.7 (0.6-0.9)*	0.7 (0.5-0.9)*	0.7 (0.6-0.8)*
Crown-rump length (cm)	1.2 (0.9-1.4)	1.2 (1-1.4)	1.1 (1-1.3) *	1.1 (0.9-1.5) *	1.1 (0.9-1.4) *

Data are shown as median (min- max).

* : p≤0.001 when compared to sham and control groups (1^st^, 2^nd^ and 3^rd^ groups were injected 10 mg/kg/day of MA respectively, from GD1 to GD7, GD8 to GD14 and GD1 to GD14).

## Discussion

In the present study, we examined the effects of prenatal MA exposure on mother and fetus in mice which were exposed during different pregnancy period. Our study showed that abuse of MA caused lower body weight gain, crown-rump length, head and also placenta circumference of fetus when the mother received 10 mg/kg/day of MA from GD1 to GD14 (3^rd^ group). Also histological changes such as exencephaly and hemorrhage were observed in this group. 

While new studies regarding the effects of MA abuse on human have been published, there is limited information about the exact mechanism on fetus development. MA abuse is known as highest illicit drug and has different aspects on both mother and fetus (18, 19). Increasingly MA consumers during pregnancy put more fetus and children at risk of developmental outcome (20). Anorexia and blood pressure are increased by misuse of MA in mother, while may decrease utero-placental blood ﬂow and cause fetal hypoxia (21). These effects could alter fetal brain development and decrease fetal growth (15). After one week, MA abuse can decrease food intake and appetite, and finally poor nutrition may affect pregnancy outcomes (22). The Nguyen *et al* investigated the effects of prenatal MA abuse on human from birth to three years old. They realized decrease in height growth but they found no significant difference in weight and head circumference during three years (19). 

Low body weight gain was reported after MA prenatal exposure in fetus that might be due to increase in the metabolism and decrease in appetite after MA administration (23). Cappon *et al* elucidated that mother and fetus have lower body weight gain compared to control when exposed to MA during pregnancy in rat, and also in another study, Cui et al showed that children whose mothers were administered MA during pregnancy had lower body weight gain (24, 25). It was shown that administration of 10 mg/kg MA can decrease body weight of fetus more than when 5mg/kg MA was treated and controls, while body weight of mothers showed insignificant difference which is similar to our results (26). The present study showed fetus and placenta weight were decreased in third group compared to others. 

MA caused high blood pressure via vasoconstriction and brain hemorrhage is reported among the people who are abusing MA (27, 28). Also administration of 5 mg/kg MA caused hemorrhage in cerebral cortex and subependymal zone in rats (27). From histological perspective, our data showed the presence of hemorrhage in second and third groups which was in line with Mirjalili *et al* study. They reported subarachnoid hemorrhage in fetuses that were exposed to 10 mg/kg and 5 mg/kg (26). Data showed prenatal exposure to 5 mmol/day of MA on GD12.5 in rat embryos significantly reduces crown rump length and somatic cells, while 5 and 10 mg/kg MA administration did not show significant difference in fetus crown-rump length on GD14 (12, 26). 

MA can cross the placenta during pregnancy (9). Previous studies have shown that MA affects CNS by elevating cytosolic catecholamines (5). Increase of catecholamines, causes a reduction of dopamine and norepinephrine nerve terminals. Their reactive metabolites produce free radicals that result oxidative DNA damage and apoptosis (29). It was showed a single dose of prenatal MA administration (20 or 40 mg/kg) leads to oxidative DNA damage and affects the brain development of fetus (16). Cui *et al* suggested interplay of MA with monoaminergic neurotransmitter systems alters development of fetal brain (25). CNS is susceptible to free radicals because of its low anti-oxidant capacity, high administration of oxygen and metabolic rate (30). 

Mirjalili *et al* reported that there is no significant difference in number of apoptotic cells in striatum region between treated groups (5 and 10 mg/kg MA) and the controls (26). Our data demonstrated more apoptotic cells that were observed in second and third groups but there were no significant difference with first and control groups (Figure 6). Oxidative DNA damage results in mutations and transcriptional delay that may lead to retardation in function and development of fetus (8). We got that that head circumference significantly decreases in first, second and third groups compared to control group. Some previous reports also have shown a reduction of head circumference in prenatally MA-exposed pregnant women (11, 12, 31).

## Conclusion

In conclusion, our data showed that MA exposure may cause lower body weight gain and placenta circumference in mouse fetus. Also MA application in utero can lead to histological alterations and induction of apoptosis by the probable toxic effects. Many questions remain to be investigated and more studies are required to find the exact mechanism of detrimental effect of prenatal exposure of MA.
